# Maternal under-nutrition during pregnancy alters the molecular response to over-nutrition in multiple organs and tissues in nonhuman primate juvenile offspring

**DOI:** 10.1017/S2040174424000163

**Published:** 2024-11-07

**Authors:** Laura A. Cox, Sobha Puppala, Jeannie Chan, Angelica M. Riojas, Kenneth J. Lange, Shifra Birnbaum, Edward J. Dick, Anthony G. Comuzzie, Mark J. Nijland, Cun Li, Peter W. Nathanielsz, Michael Olivier

**Affiliations:** 1 Center for Precision Medicine, Wake Forest University School of Medicine, Winston-Salem, NC, USA; 2 Southwest National Primate Research Center, Texas Biomedical Research Institute, San Antonio, TX, USA; 3 Department of Radiology, University of Texas Health Science Center, San Antonio, TX, USA; 4 Southwest Research Institute, San Antonio, TX, USA; 5 The Obesity Society, Rockville, MD, USA; 6 University of the Incarnate Word, San Antonio, TX, USA; 7 Department of Animal Sciences, University of Wyoming, Laramie, WY, USA

**Keywords:** Developmental programming, nonhuman primate, intrauterine growth restriction, western diet

## Abstract

Previous studies in rodents suggest that mismatch between fetal and postnatal nutrition predisposes individuals to metabolic diseases. We hypothesized that in nonhuman primates (NHP), fetal programming of maternal undernutrition (MUN) persists postnatally with a dietary mismatch altering metabolic molecular systems that precede standard clinical measures. We used unbiased molecular approaches to examine response to a high fat, high-carbohydrate diet plus sugar drink (HFCS) challenge in NHP juvenile offspring of MUN pregnancies compared with controls (CON). Pregnant baboons were fed *ad libitum* (CON) or 30% calorie reduction from 0.16 gestation through lactation; weaned offspring were fed chow *ad libitum*. MUN offspring were growth restricted at birth. Liver, omental fat, and skeletal muscle gene expression, and liver glycogen, muscle mitochondria, and fat cell size were quantified. Before challenge, MUN offspring had lower body mass index (BMI) and liver glycogen, and consumed more sugar drink than CON. After HFCS challenge, MUN and CON BMIs were similar. Molecular analyses showed HFCS response differences between CON and MUN for muscle and liver, including hepatic splicing and unfolded protein response. Altered liver signaling pathways and glycogen content between MUN and CON at baseline indicate *in utero* programming persists in MUN juveniles. MUN catchup growth during consumption of HFCS suggests increased risk of obesity, diabetes, and cardiovascular disease. Greater sugar drink consumption in MUN demonstrates altered appetitive drive due to programming. Differences in blood leptin, liver glycogen, and tissue-specific molecular response to HFCS suggest MUN significantly impacts juvenile offspring ability to manage an energy rich diet.

## Introduction

In 2021, the United States Department of Agriculture (USDA) revealed that 12.5% of US households with women of reproductive years and children experienced food insecurity.^1^ Poor maternal nutrition during pregnancy is linked to intrauterine growth restriction (IUGR) and adverse lifecourse health outcomes in offspring. Human epidemiological studies and controlled nutrient reduction studies in rodents and sheep show that a suboptimal intrauterine environment leading to IUGR alters the trajectory of fetal development with profound effects on life-time offspring health (reviewed in).^2^ IUGR is strongly associated with heart disease, hypertension, obesity, and diabetes in adult offspring of these suboptimal pregnancies. For example, rats fed low-protein diets have high blood pressure from early postnatal life.^3^ In addition, offspring of low-protein fed dams have increased susceptibility to diabetes, insulin resistance, and hypertension when fed a high-fat diet.^4^ similar to what has been observed in human epidemiological studies.^5^

To date, studies of mechanisms by which maternal under nutrition (MUN) during pregnancy programs offspring health have been conducted primarily in rodents making it difficult to extrapolate to humans due to significant developmental (and nutritional) differences between rodents and nonhuman primates (NHP).^6^ For example, at birth several rodent organs such as kidney are developmentally similar to mid-gestation stages in primates due to the need for rodent organs to be functional at birth.^7^ This difference alters the trajectory of rodent organ development compared with primates. These differences are apparent for both cellular and molecular properties.^8^ The NHP model of MUN leading to IUGR offers the advantages of defining offspring phenotype and mechanisms using a model that is physiologically and genetically similar to humans and for which the environment can be controlled.

In previous studies, we showed in NHP that MUN (70% of total calories fed *ad libitum* to control (CON) dams) during pregnancy results in IUGR in both female and male offspring.^9^ We demonstrated that MUN affected the transcriptome and energy storage in the developing fetal liver via epigenetic changes in phosphoenolpyruvate carboxykinase signaling.^10,11^ In addition, we found effects on the fetal liver transcriptome through increased RNA splicing and potential metabolic alterations with increased enrichment of genes for fatty acid beta oxidation signaling.^12^ Abundance of orexigenic neuropeptide Y was increased, anorexigenic proopiomelanocortin was decreased, and leptin signaling down-regulated in the hypothalamus of 0.9 gestation IUGR fetuses,^13^ consistent with reduced fetal nutrition programing offspring for increased appetite and later life obesity. These changes are similar to those shown in rodent models where gene expression for orexigenic peptides increases with corresponding decreases in gene expression for anorexigenic peptides in hypothalamic arcuate (ARH) nuclei of IUGR offspring.^14^ These hypothalamic peptide and leptin feedback signaling changes suggest that MUN resulting in IUGR alters energy sensing pathways and increases appetite. Consistent with these findings, studies in pre-pubertal MUN baboons show altered peripheral insulin sensitivity, potentially predisposing these animals to early onset of type 2 diabetes.^15^

Based on our studies of MUN effects on the NHP fetus, we hypothesized that fetal programing by MUN persists postnatally, which alters the molecular response to the dietary mismatch of an energy dense diet, and that these molecular signaling alterations in metabolic systems precede alterations in standard clinical metabolic measures. Due to rodent-primate genetic differences in organ development and for identification of molecular systems altered by developmental programing in primates, we thought it is important to use an unbiased approach to address our hypothesis because targeted measures based on rodent studies would potentially miss important molecular changes.

Our study included MUN juvenile baboons aged ~ 4.5 years old and age-matched CON that were maintained on a low cholesterol, low fat diet from weaning until study initiation. We assessed whole body, cellular, and molecular responses before and after a 7-week *ad libitum* high-fat, high-carbohydrate, high-salt Western style diet supplemented with a high fructose drink (HFCS).

We found that fetal programing persists in NHP offspring with significant differences between juvenile MUN and CON offspring before the diet challenge. Interestingly, the tissue-specific response to the HFCS challenge, in both the numbers of genes and molecular pathways impacted, emphasizes the dramatic differences across multiple tissues in MUN offspring compared to CON animals when fed an energy dense diet. Our findings demonstrate that *in utero* exposure to MUN alters molecular pathways and regulatory mechanisms that likely lead to major changes in appetite, and energy management and storage in MUN juvenile offspring.

## Methods

### Ethics statement

All animal procedures and study protocols were approved by the Institutional Animal Care and Use Committee at Texas Biomedical Research Institute and conducted in Association for Assessment and Accreditation of Laboratory Animal Care approved facilities at the Southwest National Primate Research Center.

### Animal selection and management

Baboon housing, feeding, and environmental enrichment were previously published.^16^ All dams spontaneously delivered offspring at full term. Offspring were reared with their mothers in group housing until weaning at ~ 9 months, and then maintained on chow diet until diet challenge.

### HFCS diet challenge and sugar drink consumption

**Figure 1.**
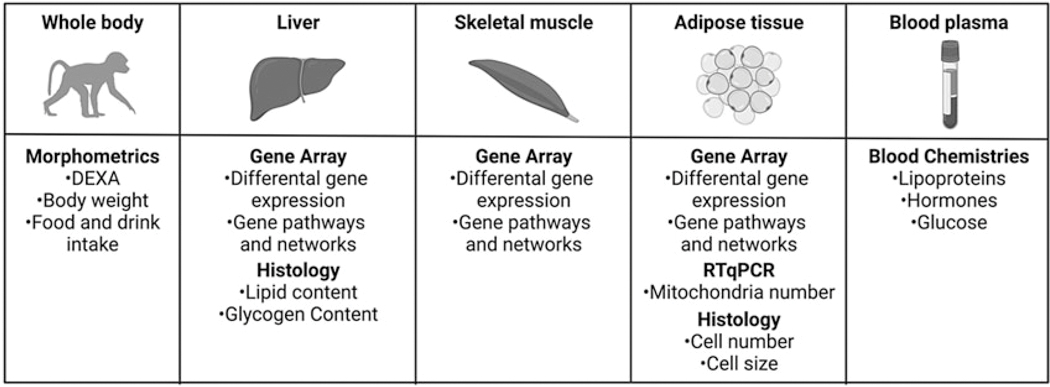
Overview of study design. Offspring investigated in this study were juvenile age from differing maternal in utero environments prior to a 7-week high fat, high fructose, high-salt diet with high fructose drink data was collected from whole body, liver, skeletal muscle, adipose, and blood plasma for functional and molecular analysis. Created with BioRender.com.

At ~ 4.5 years of age (human equivalent 13 years), 6 CON offspring (3 females, 3 males) and 6 age-matched MUN offspring (3 females, 3 males) were challenged with a 7-week *ad libitum* HFCS diet, high fructose drink with free access to water (Fig. 1A). HFCS diet and high fructose drink details were previously published.^17^

Baboons ran once per week into individual feeding cages, passing over an electronic weighing scale, and fed *ad libitum* HFCS diet for 16 hours. High fructose drink and water were provided in Lixit waterers (Lixit, Napa, California) with gauges to measure consumption for each animal, and consumption adjusted to each animal’s body weight.

### Health assessment and morphometric measurements

Animal weights were obtained weekly.^16^ Morphometric measurements were collected at baseline and end of challenge. Baboons were sedated with 10 mg/kg Ketamine Hydrochloride (Ketaset, Iowa) administered intramuscularly. Hair was removed around the waist and hip circumference lines for accurate measurements. Body length (recumbent length) was measured using an anthropometer (Catalog #N101, SiberHegner Ltd Switzerland) from head crown to right tibia. Anterior-posterior abdominal distance was measured from the plane of the back to the anterior point of the abdomen at the level of navel. Waist circumference was measured mid-way between the lowest point of the ribs in the mid-axillary line (costal margin) (10^th^ rib) and iliac crest in the mid-axillary line. Hip circumference measurements at the point of maximum circumference over the buttocks. Body Mass Index (BMI) was calculated by dividing the weight in kilograms by the crown-rump length in cm^2^ (Figure 1B).

### Blood and biopsy collection:

Blood and tissue biopsies from liver (right lobe), skeletal muscle (vastus lateralis), and adipose (omental) were collected at baseline and after the 7 week challenge. Animals were fasted overnight and immobilized with inhalation anesthesia with isoflurane (1.5%, v/v), and percutaneous venipuncture performed on the femoral vein just caudal to the femoral triangle. A 10 mL vacutainer tube was drawn for serum and a 10 mL Na_2_EDTA vacutainer tube was drawn for plasma. Plasma samples were assayed in duplicate. Needle biopsies of 25 mg liver, 5 mg muscle, and 1 gm omental fat were collected from sites aseptically prepared and locally infiltrated with lidocaine. Tissues were placed in tubes, snap frozen in liquid nitrogen, and stored at −80°C.

### Body composition (DEXA scans)

Body composition was measured by dual-energy X-ray absorptiometry scan (DEXA; Lunar Prodigy; GE Medical Systems, Madison, WI). Animals were placed in supine position on the DEXA bed and extremities positioned within the scanning region. Scans were analyzed using encore2007 software version 11.40.004 (GE Healthcare, Madison, WI). Total body, torso, and each arm and leg, waist, hip region compositions were determined.

### Histological analysis of liver and omental adipose biopsies

Frozen fat and liver samples were simultaneously defrosted and fixed in cold formalin, and processed using graded alcohols, xylene, and paraffin embedded. Paraffin blocks were cut in 3 mm sections and processed simultaneously to ensure consistency. Details are described previously (PMID: 19574404).

Liver sections were stained with Oil Red O to quantify lipid content (Abcam, Catalog #ab150678) and separate sections with Periodic acid Schiff (PAS) to quantify glycogen content (Abcam, Catalog #ab150680). Images of sections were captured at 10x magnification using an Olympus BX-41 microscope and QImaging QIcam 12-bit Fast 1394 camera with Bioquant Osteo 2013 software. Three slides per animal per diet were analyzed with six pictures (2650 × 1920 pixels) taken from each slide at 2, 4, 6, 8, 10, and 12 o’clock positions and analyzed using NIH Image J version 1.49d software.^18^ Analyses included measuring fraction (area of positive stained × 100%) for Oil Red O, and density (in arbitrary density units) for PAS.

Adipose tissue sections were stained with hematoxylin and eosin, and adipocytes counted using the multipoint tool in Image J. Freehand selection and brush selection tools were used to obtain areas of the field of view without adipocytes. Sums of non-adipocyte areas were subtracted from the total image area to obtain the area of adipocytes. Adipocyte area was divided by total number of adipocytes to obtain average adipocyte size for each field of view. To convert pixel area obtained from Image J into micrometers, each area was multiplied by pixel size squared (0.769231^2^ = 0.59171633).^19^

### Skeletal muscle mitochondrial number

Genomic DNA was extracted from skeletal muscle biopsies according to the manufacturer’s protocol (Qiagen DNA Purification Kit). Quantitative PCR (qPCR) was performed by SYBR green PCR Kit (Thermo Fisher) with baboon endothelial lipase exon 5 specific primers as the single gene endogenous control (Forward, 5’-TGCACACCCAGGCTTAACTTGT-3’; and Reverse, 5’-CCCAAGACATCGTTGAGTCCAC-3’) and baboon mitochondrial sequence specific primers: (M16SForward, 5’-GCAAACCCTGATGAAGGCTA-3’; and M16SReverse, 5’-GGCCCTGTTCAACTAAGCAC-3’) with 10 ng of DNA per sample run in triplicate, and amplification and quantification using an ABI7900 Real Time PCR Instrument. A pooled baboon DNA sample as positive control and a no template sample as negative control were included, each in triplicate.

### Plasma and serum measures

Fasting plasma concentrations of glucose (Alfawassermann Cat. #293598), alanine aminotransferase (ALT; Alfawassermann Cat. #275454), aspartate aminotransferase (AST; Alfawassermann Catalog #282242), triglyceride, total cholesterol, LDL, and HDL were determined using an ACE clinical analyzer.^20^ Total hemoglobin (THB) and percent HbA_1c_ were determined from whole blood using an ACE clinical analyzer.^17^ Cortisol was measured by Immulite assay (Siemens/DPC Catalog #914038), insulin by Immulite assay (Siemens/DPC Catalog #914047), adiponectin by Immulite assay (EMD Millipore Catalog #HADK1MAG-61K), and leptin by Radioimmunoassay (RIA; EMD Millipore, Catalog #HL-81K).

### Differential gene expression

Total RNA was isolated from liver, adipose and muscle biopsy samples, quality checked, complementary DNA (cDNA) synthesized, and biotinylated as described,^8^ using HumanHT-12 v4 Expression BeadChips (Illumina, Inc.).^21^ Gene expression data were extracted and log_2_-transformed using GenomeStudio software (Illumina, Inc.) and analyzed using Partek^®^ Genomics Suite (Partek^®^, St Louis, MO). Principal Component Analysis and hierarchical clustering in Partek^®^ Genomics Suite identified treatment group and diet as the greatest source of variation in each dataset, but no significant contributions by sex for any tissues; therefore, we combined female and male data for analyses. Signal intensities were quality filtered (>0.95), quantile normalized, and differentially expressed genes (DEG) identified by Analysis of Variance (ANOVA; FDR p-value < 0.05, Fold change > 1.2).

### Pathway Enrichment Analysis:

DEG (p-value < 0.05) were overlaid onto canonical pathways using Ingenuity Pathway Analysis (IPA; QIAGEN) Knowledge Base. Right-tailed Fisher’s exact test was used to calculate enrichment of DEG in pathways, *p* < 0.01.^22^ Pathways with –log p-value > 1.3 (p < 0.01) that also contained one or more DEG FDR p-value < 0.05 were considered significant. Pathways were considered biologically coordinated if gene expression directionality at the end-of-pathway (EoP) was consistent with overall pathway directionality.^23^

### Regulatory network analysis

Upstream regulatory network analysis was performed with Z-scores predicting regulatory directions and inferring activation/inhibition state of a putative regulator. Detailed statistical methods are provided.^24^ Networks were built using the IPA Knowledge Base and required p-values < 0.01, direct connections between molecules based on experimental evidence, differential expression of network regulators was consistent with activation/inhibition status of the networks, downstream targets were differentially expressed, and networks containing at least one FDR DEG (FDR p-value < 0.05) were considered significant. Networks were considered biologically coordinated if manual annotation showed gene expression directionality was consistent with overall network directionality.^23^

### Statistical analyses for morphometric, clinical, and histological data

As with gene array data, we found no differences between females and males for morphometric, clinical, or histological datasets; therefore, we combined females and males for all analyses. Weight adjusted fructose consumption was calculated by dividing 7-week average fructose consumption by 7-week average body weight. Pairwise comparisons were performed using two tailed *t*-tests. Data from each diet were analyzed independently using two-way ANOVA. Statistical significance was adjusted for multiple testing with an adjusted *p*-value < 0.05. Regression analyses for fat cell size and adipokine measures were performed using GraphPad Prism (graphpad.com).

## Results

This study included whole animal, hormonal, cellular, and molecular characterization of juvenile MUN offspring of a unique NHP model of developmental programing and matched CON animals (Figure 1A,B). Our analysis compared animals before and after a 7-week *ad libitum* HFCS Western style diet supplemented with a high fructose drink (Figure 1A).

### Comparison of MUN versus CON at baseline

#### Morphometrics

MUN offspring weighed less than CON at birth. At the beginning of the study (baseline), age-matched CON and MUN offspring were the same height; MUN weighed less and had lower BMI (Table 1), suggesting continued impact of MUN during pre-pubertal development. Body composition showed MUN offspring had less fat mass and total mass, and marginally less lean mass than CON (Table 2).

#### Lipoprotein and hormonal measures

Plasma measures showed AST, ALT, cortisol, glucose (Table 3), HbA1c, and THB (data not shown) were not different between CON and MUN at baseline. Also, no differences were observed in LDL, HDL, total cholesterol, and triglycerides at baseline (Table 4). Leptin, and adiponectin levels were higher in MUN than CON (Table 5).

### Molecular analyses of liver, skeletal muscle, and omental adipose tissues

We found the greatest differences between MUN versus CON at baseline in liver with 247 DEG, 194 up-regulated and 53 down-regulated (Table 6, Table S1). Skeletal muscle revealed 84 DEG, 49 up-regulated and 35 down-regulated (Table 6, Table S2). Adipose revealed only one DEG which was up-regulated in MUN compared with CON (Table 6, Table S3).

Pathway enrichment analysis reflected DEG numbers with the greatest number of pathways in liver and then skeletal muscle (Table 7). Of the 46 liver pathways, 6 met the EoP criteria; notably, spliceosomal cycle (Figure S1), unfolded protein response (UPR) (Figure S2), and BAG2 signaling (chaperone folding activity) (Figure S3) were up-regulated (Table S4). Skeletal muscle pathways meeting EoP criteria included up-regulation of oxidative phosphorylation (Figure S4), nucleotide excision repair, and estrogen signaling, with down-regulation of calcium signaling (Figure S5) and beta adrenergic signaling (Table S5). Enriched adipose tissue pathways did not include any genes meeting FDR criteria, and of 17 pathways, only ILK signaling met EoP criteria (Table S6).

Network analysis, similar to pathway analysis, showed the greatest differences in liver with 4 networks (Table 8). One network regulated by KLF6 was inhibited and contained 20 target genes; 75% of the targets’ expression profiles were consistent with network inhibition (Figure S6, Table S7). Three networks were activated by XBP1 (X-Box Binding Protein 1), a transcription factor that regulates UPR, YAP1 (Yes1 Associated Transcriptional Regulator), a nuclear effector of growth, repair, and homeostasis, and TP63 (Tumor Protein P63), a transcription factor, These networks included a total of 178 gene targets, with some common among the three networks, and 25% of the targets’ expression profiles were inconsistent with network activation (Figure S7, Table S7). Skeletal muscle revealed one network regulated by DDX5 (DEAD-Box Helicase 5), a RNA helicase that coregulates mRNA processing; and 10 of the 12 (83%) target gene expression profiles were consistent with network activation (Figure S8, Table S8). No networks meeting filtering criteria were found in adipose (Table S9).

### Cellular analyses of liver, skeletal muscle, and omental adipose tissues

**Figure 2.**
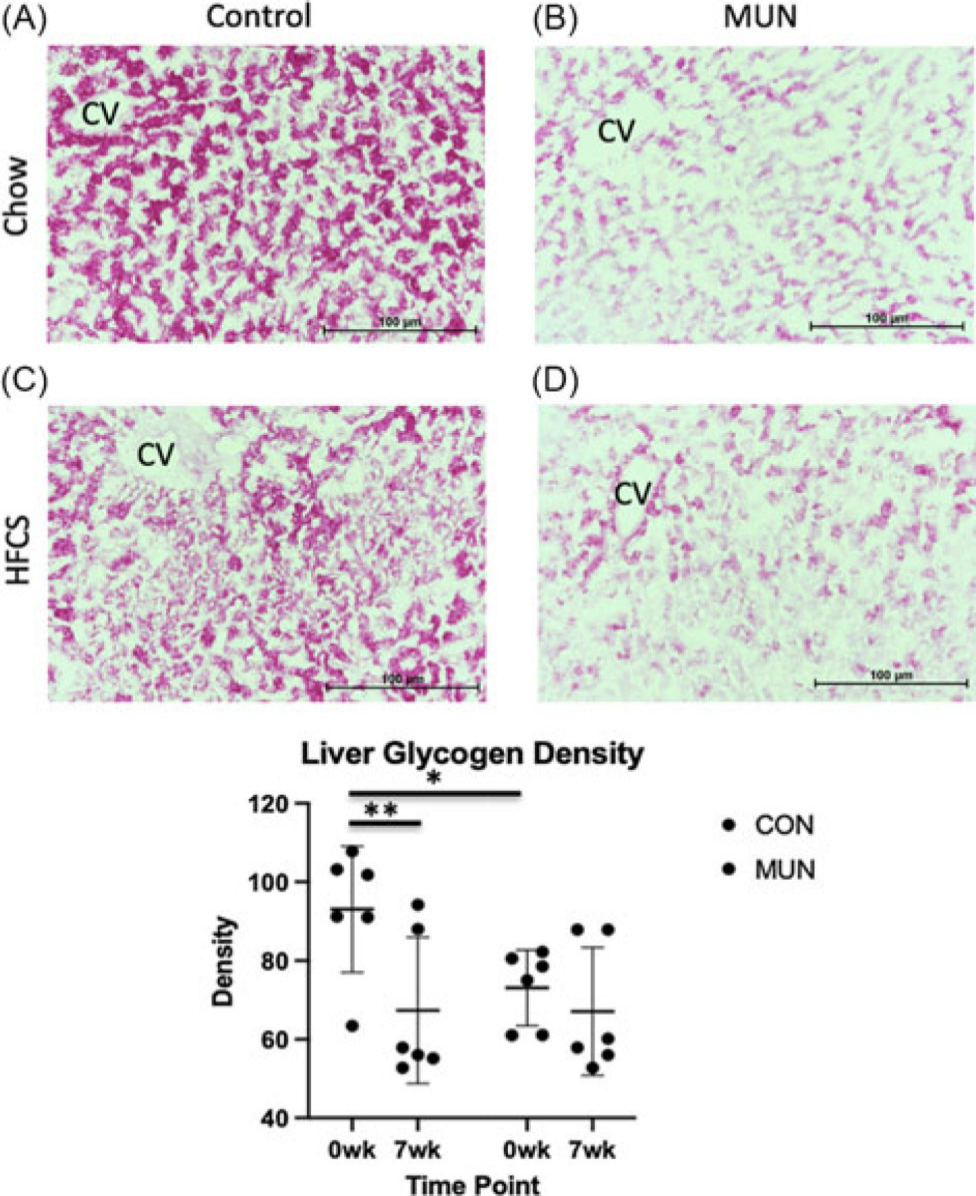
Liver glycogen density from control (CON) (A) and maternal undernutrition (MUN) (B) at baseline, CON (C) and MUN (D) after a 7-week high fat, high fructose, high-salt diet with high fructose drink. E. shows results for CON (*n* = 7) and MUN (*n* = 6) at both time points. Circles indicate CON and boxes indicate IUGR. * denotes adjusted *p* < 0.05.

**Figure 3.**
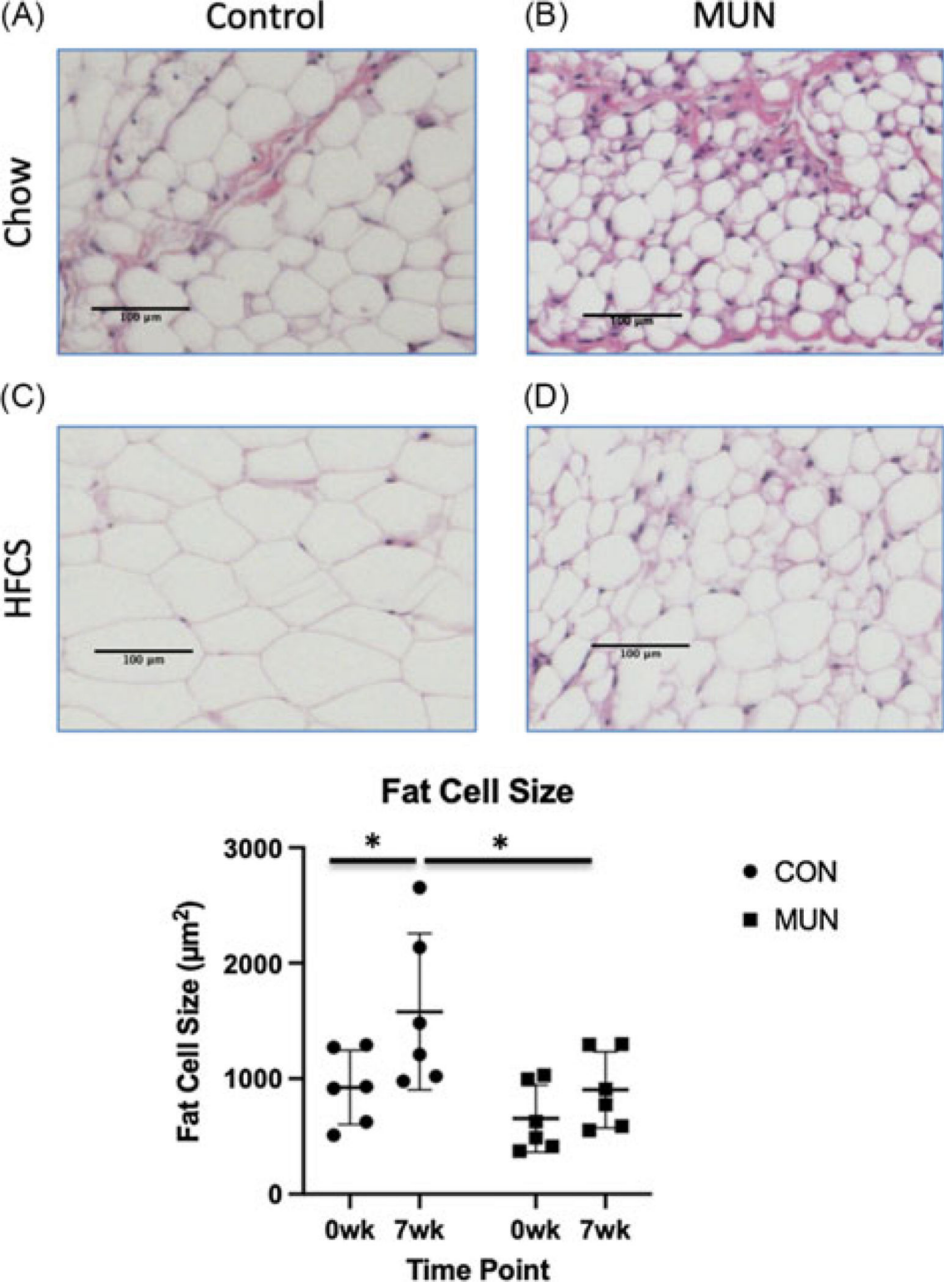
Adipocyte size in representative omental fat sections from control (CON) (A) and maternal undernutrition (MUN) (B) at baseline, CON (C) and MUN (D) after a 7-week high fat, high fructose, high-salt diet with high fructose drink. E. shows results for CON (*n* = 7) and MUN (*n* = 6) at both time points. Circles indicate CON and boxes indicate MUN. * denotes adjusted *p* < 0.05.

Due to limited amounts of biopsy material available for cellular analyses of tissues, we focused on phenotypes most relevant to each tissue. Histological analysis of liver showed less glycogen in MUN versus CON (Figure 2); however, no differences in lipid content were observed (not shown). Histological analysis of adipose tissue showed fat cell size did not differ (Figure 3), and qPCR of skeletal muscle mitochondria showed no differences between MUN and CON (not shown).

### Response to HFCS diet challenge in MUN and CON

To assess whether MUN programs dyslipidemia, diabetes, and appetite in primates, we studied response to a HFCS diet supplemented with a high fructose drink. We monitored drink consumption as an indication of appetitive drive, and quantified changes in body composition and morphometrics, metabolism, liver function, molecular effects, and cellular effects of the challenge.

#### Morphometrics and fructose drink consumption

Both groups increased in height from baseline to the end of the 7-week challenge. Although weight and BMI were lower in MUN than CON at baseline, they did not differ at the end of challenge (*p* < 0.05) (Table 1). In addition, body composition measures of fat mass, lean mass, and total mass did not differ between MUN and CON at the end of the challenge (Table 2), indicating more rapid growth in MUN than CON animals during the challenge. Average sugar drink consumption over 7 weeks, adjusted for each animal’s weight, was approximately 70% greater in MUN versus CON offspring (p < 0.01) (Table 9).

#### Lipoprotein and hormonal measures

Plasma adipsin increased and leptin decreased in both CON and MUN animals in response to HFCS challenge. Cortisol (Table 3), adiponectin, glucose, and insulin did not change (Table 5). Plasma ALT (Table 3) and HDL (Table 4) decreased, and triglycerides (Table 4) increased in CON but not MUN; whereas AST (Table 3), LDL and TSC (Table 4) decreased only in MUN in response to the challenge.

#### Liver, skeletal muscle, and adipose transcriptomes

Transcriptome response to the challenge differed markedly by tissue in MUN versus CON. For CON animals, the greatest response was in skeletal muscle with 332 DEG, 195 up-regulated and 137 down-regulated. In contrast, MUN animals’ greatest response was in liver with 909 DEG, 295 up-regulated and 614 down-regulated. Far fewer DEG were identified in adipose, but it is worth noting that the number of DEG (FDR<0.05) in CON adipose in response to the HFCS diet was greater than in MUN (CON = 36, IUGR = 8) (Table 10, Supplemental Tables 1 – 3).

Pathway analysis for CON liver response to HFCS diet showed 8 pathways with 4 meeting EoP criteria, all related to cholesterol biosynthesis and down-regulated (Figure S9, Table S4), indicating a coordinated response to the HFCS diet in CON animals. In MUN livers, HFCS diet response identified 81 pathways that met FDR DEG requirements, but of these, only one met EoP criteria - dolichyl-diphosphooligosaccharide biosynthesis - indicating lack of coordinated response to the challenge in livers of MUN animals (Table 11, Table S4). For CON skeletal muscle response, 2 of 24 significant pathways met EoP criteria, oxidative phosphorylation (Figure S10) and glycolysis I signaling, both were down-regulated. In MUN skeletal muscle HFCS response, 3 of 6 pathways met EoP criteria – oxidative phosphorylation (Fig. S11), calcium signaling, and TCA cycle signaling, all were down-regulated. Oxidative phosphorylation in skeletal muscle was the only down-regulated pathway common to CON and MUN HFCS response (Table 11, Table S5). In adipose tissue, no pathways met filtering criteria for either CON or MUN (Table 11, Table S6).

Network analysis in liver of CON animals’ response to HFCS revealed 7 networks were inhibited by NFKBIA (NFKB Inhibitor Alpha), an NFKB inhibitor, FOXO1 (Forkhead Box O1), a transcription factor, STAT3 (Signal Transducer And Activator of Transcription 3), a mediator of cellular responses to growth factors, CEBPB (CCAAT Enhancer Binding Protein Beta), a basic leucine zipper domain containing transcription factor, SREBF1 (Sterol Regulatory Element Binding Transcription Factor 1), a basic helix-loop-helix-leucine zipper transcription factor, NUPR1 (Nuclear Protein 1, Transcriptional Regulator), a transcriptional coactivator, and NR5A2 (Nuclear Receptor Subfamily 5 Group A Member 2), a DNA binding zinc finger transcription factor. These networks included a total of 68 targets with 82% showing expression profiles consistent with inhibitory networks (Figure S12, Table 12, Table S7). MUN livers showed 4 networks inhibited by XBP1 (X-Box Binding Protein 1), an X-box binding transcription factor, ATF4 (Activating Transcription Factor 4), a transcription factor that binds the cAMP response element, NUPR1 (Nuclear Protein 1, Transcriptional Regulator), a transcriptional coactivator, and MLXIPL (MLX Interacting Protein Like), a basic helix-loop-helix-leucine zipper transcription factor that activates carbohydrate response elements in a glucose-dependent manner. The networks for these regulators include a total of 308 targets, of which 55% were down-regulated (Figure S13, Table 12, Table S7).

In skeletal muscle, 4 regulatory networks met criteria for CON response to HFCS. Two networks were inhibited by regulators HNF4A (Hepatocyte Nuclear Factor 4 α), a transcription factor, and SMAD7 (SMAD Family Member 7), a signaling antagonist, with 60% of the 221 targets were down-regulated (Figure S14, Table 12, Table S8); and 2 networks were activated by regulators ESR1 (Estrogen Receptor 1), a ligand activated transcription factor, and IGF2BP1 (Insulin Like Growth Factor 2 mRNA Binding Protein 1), an RNA binding factor that facilitates mRNA transport, but only ~ 50% of the targets were up-regulated (Figure S15, Table 12, Table S8). For MUN skeletal muscle network response to HFCS, one network regulated by PPARGC1A (PPARG Coactivator 1 α), a transcriptional coactivator, was inhibited with most targets down-regulated (Figure S16, Table 12, Table S8).

In adipose tissue, CON HFCS response revealed one network activated by ESR1 and contained 76 targets of which 61% were up-regulated. This network is of interest in that it contains genes related to wound healing, phagosome formation, glucocorticoid signaling, and G-protein coupled receptor signaling. Also of interest is inclusion of ACACA (Acetyl-CoA Carboxylase Alpha), the rate limiting enzyme in fatty acid synthesis, which has not previously been linked to adipose tissue metabolic signaling (Figure S17). In MUN adipose tissue, HFCS response revealed one network inhibited by SREBF2 (Sterol Regulatory Element Binding Transcription Factor 2), a transcription factor that regulates cholesterol homeostasis, and one network activated by EPAS1 (Endothelial PAS Domain Protein 1), a transcription factor induced by oxygen. In the SREBF2 network, only 7 of the 15 (47%) target genes were down-regulated (Figure S18), and in the EPAS1 network, 50% of the 16 target genes were up-regulated. Interestingly, both networks contained genes related to sirtuin signaling, glucocorticoid signaling, TR/RXR activation, and LXR/RXR activation (Figure S19, Table 12, Table S9). These findings in MUN adipose were similar to liver pathway enrichment in which only one of the 81 pathways met EoP criteria suggesting lack of coordinated molecular response to HFCS challenge in MUN offspring.

#### Cellular response to the HFCS challenge: liver, skeletal muscle, and omental adipose tissues

Liver glycogen content decreased from baseline to the end of challenge in CON, but not in MUN offspring (Figure 2). Adipocyte size increased in CON from baseline to the end of challenge and was greater than MUN at the end of challenge; while MUN adipocyte size did not change from baseline to the end of challenge (Figure 3). Interestingly, adiponectin, leptin, and adipsin measures did not correlate with adipocyte cell size for either group on either diet (not shown). Mitochondrial number in skeletal muscle did not differ between MUN and CON (not shown).

## Discussion

A common scenario in many human populations is exposure to a poor nutritional environment *in utero* followed by a postnatal calorie rich environment. This mismatch in available calories is thought to predispose MUN offspring to early onset metabolic dysregulation leading to obesity,^25^ type 2 diabetes,^26,27^ and cardiovascular disease.^28,29^ Previous studies have shown that moderately reduced caloric intake in our baboon model detrimentally impacts overall fetal growth leading to IUGR,^30^ with alterations to the fetal heart,^31,32^ adipose,^33^ kidney,^23,34,35^ liver,^10,36^ and brain frontal cortex.^37^ In addition, IUGR correlates with changes in abundance of appetitive neuropeptides in the ARH of the near-term fetus, which may alter appetitive drive and contribute to obesity in MUN offspring.^9^

Two major questions from NHP fetal studies are: 1) Do fetal effects of MUN persist in postnatal life of IUGR juvenile offspring? 2) Does a caloric mismatch between *in utero* and postnatal life result in appetitive and metabolic dysregulation in juvenile primates? To our knowledge this is the first study comparing CON and MUN postnatal juvenile NHP, and the impact of total caloric mismatch between fetal and postnatal life on metabolic functions in CON and MUN NHP juveniles. In our studies on fetal impact of MUN, we found molecular alterations in multiple tissues that differed from those altered in rodents (e.g.,^38–41^). Therefore, we used unbiased molecular analyses to identify signaling pathways and regulatory networks altered by MUN.

To address the question whether fetal effects of MUN persist in postnatal life of IUGR NHP juvenile offspring, we compared molecular pathways, as well as morphometric measures, appetite, and clinical measures of metabolism in MUN with CON age-matched offspring prior to a HFCS challenge, i.e., animals were maintained on a low cholesterol, low fat chow diet from weaning until the study began. Liver molecular analyses revealed that few pathways containing DEG had coordinated activity, including many metabolic pathways such as fatty acid beta oxidation, oxidative phosphorylation, and mTOR signaling. In addition, pathways that showed coordinated regulation included spliceosomal cycling, UPR and BAG2 signaling, which all play roles in molecular diversity, and were up-regulated in MUN compared with CON. These findings are consistent with our previous study of MUN fetal livers in which we also found increased transcript splicing.^12^ In skeletal muscle, we found many significantly enriched pathways, however, similar to liver almost all pathways showed lack of coordinated regulation. The one coordinated pathway was calcium signaling, which was down-regulated, suggesting decreased muscle function in MUN offspring compared with CON.

**Figure 4.**
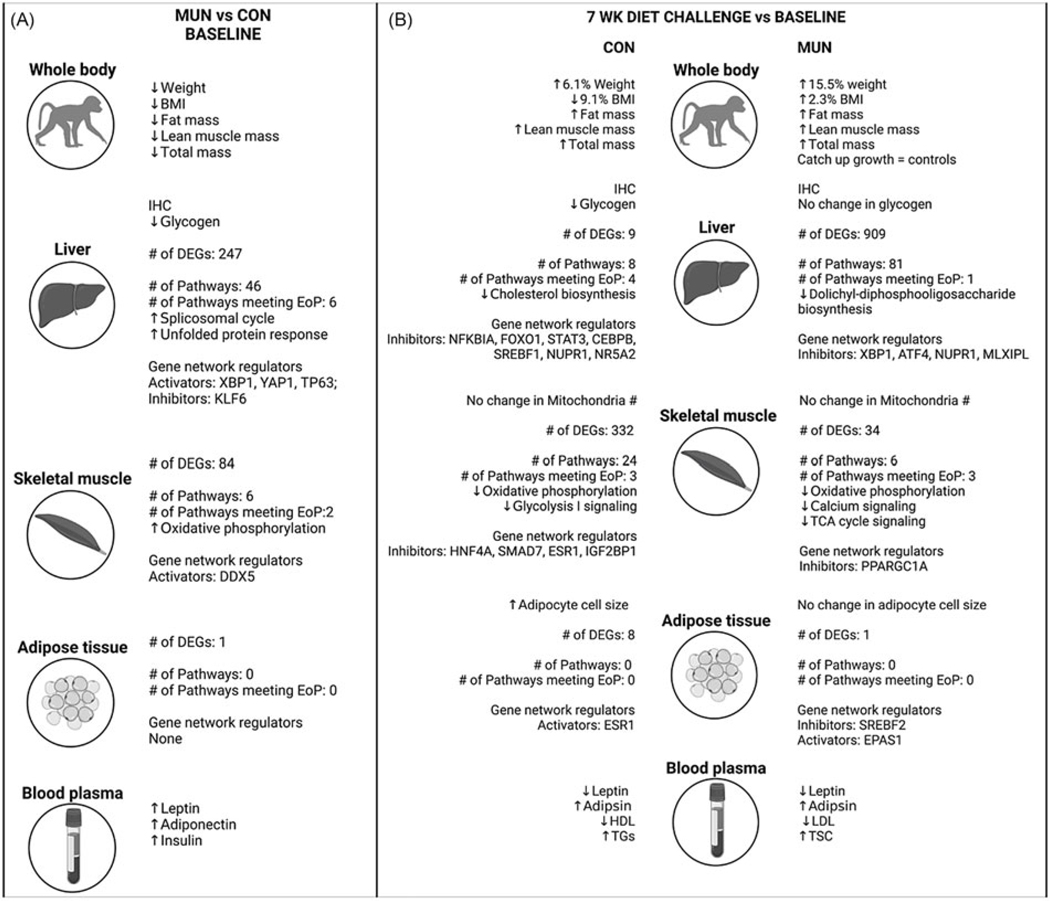
Summary of study findings. Results from comparisons in maternal undernutrition (MUN) vs control (CON) animals at baseline (A) are shown with arrows indicating a measure was statistically significantly increased or decreased in MUN animals across whole body, skeletal muscle, liver, adipose, and blood measures. Findings from gene array analysis including gene pathways and networks are also summarized. Results from the 7 week diet challenge compared to the baseline timepoints for CON and MUN groups respectively follow the same data summary organization as A. Differentially expressed genes, end of pathway. Created with BioRender.com.

At the beginning of the study, MUN animals weighed less, and had less muscle and fat mass than CON. In addition, MUN animals had greater serum concentrations of satiety hormones adiponectin and leptin, and lesser amounts of liver glycogen in MUN offspring compared with CON. Taken together, our results strongly support persistence of fetal programing in primate juvenile MUN offspring with overall growth restriction, altered appetitive drive, and altered molecular networks in metabolic tissues (Fig. 4A).

To address the question of MUN offspring response to caloric mismatch, we compared age-matched CON and MUN juvenile offspring response to a 7-week HFCS diet plus high fructose drink. The challenge aimed to mimic a mismatch of nutrients between fetal and juvenile developmental periods. MUN offspring weighed the same as CON, with no differences in body weight, fat mass, or lean mass at the end of challenge suggesting catchup growth by MUN offspring. Few studies have been published evaluating metabolism in mammalian MUN juvenile offspring that demonstrated catch-up growth. One study of IUGR juvenile sheep by Muhle et al.^42^ found catch up growth in response to an energy dense diet. Studies in humans and animal models have shown that catch-up growth in IUGR offspring is associated with higher abdominal fat mass, blood pressure, total cholesterol, and insulin resistance (e.g., ^43,44^).

Quantification of satiety hormones showed 2.6-fold greater serum concentrations of leptin in MUN versus CON offspring. These findings are consistent with increased fructose drink consumption in MUN offspring and indicate that satiety signals differ in MUN offspring compared with CON. We did not find differences in measures related to liver function, lipid metabolism, or glucose metabolism. However, we did find differences in liver glycogen content with CON animals showing decreased glycogen content in response to the challenge, with no change in MUN (Fig. 4B). Our findings are similar to Stanhope and Havel who showed association of failed insulin rise with high fructose consumption and detrimental metabolic consequences such as obesity and insulin resistance.^45^

It is possible that the effects of MUN on clinical measures related to liver function are not manifest during the juvenile developmental period due to the large metabolic demands of rapid growth. This is supported by the dramatic tissue-specific differences in HFCS diet response where CON offspring greatest response is in skeletal muscle, but MUN offspring greatest response is in liver, which is also supported by the differences in liver glycogen with CON animals but not MUN (Fig. 4B).

Our observations of uncoordinated regulation of metabolic pathways and coordinated regulation of pathways specifically related to molecular diversity, are similar to studies of environmental challenges in yeast,^46^ plants,^47,48^ invertebrates,^49^ and vertebrates,^50^ showing increased molecular diversity with different stressors. Furthermore, studies of aging have shown dysregulation of mTOR signaling, autophagy, and mitochondrial energetics which negatively impacts aging processes in organisms ranging from worms, to flies, to mice.^51^ Our findings of a large number of DEG enriching a large number of pathways that are not coordinately regulated in MUN liver and skeletal muscle suggest that the increased molecular diversity is not adaptive, but requires further investigation.

An interesting observation relevant to metabolism and energy management is increased fat cell size in CON with HFCS diet, but not MUN offspring (Fig. 4B), providing additional cellular evidence of different energy management by MUN offspring than CON, i.e., tissue-specific management of excess energy. Previous studies have reported that large adipocytes increase release of inflammatory cytokines with detrimental health outcomes.^52–54^ Although we see an increase in adipocyte size in CON offspring, cell size is still within Category I.^55^ Thus, CON offspring adipocyte size increase may not be metabolically detrimental, but rather a healthy response to a high-energy diet in juveniles, i.e., proper storage of excess lipid. In contrast, the lack of adipocyte size increase and greater proportion of small adipocytes in MUN offspring, which have been associated with ectopic lipid accumulation,^56^ suggest a maladaptive response to the high-energy diet. Our findings of fundamental adipocyte differences are supported by molecular data showing coordinated networks in CON but not MUN offspring. In addition, the ESR1 regulated network in CON adipose includes ACACA, the rate limiting enzyme in fatty acid synthesis,^57^ a gene not previously associated with adipose energy management. Our unbiased molecular analyses may have revealed ACACA as an important factor influencing adipocyte function. Taken together, our study reveals fundamental differences between MUN and CON offspring when fed a healthy chow diet, as well as significantly different responses to the HFCS diet at the hormonal, cellular, and molecular levels.

## Conclusion

We hypothesized that programing by MUN persists postnatally, and that molecular signaling alterations in metabolic systems precede alterations in standard clinical metabolic measures. We found that MUN juveniles challenged with an energy dense diet for 7 weeks, a mismatch from the *in utero* environment, demonstrated tissue-specific molecular signaling differences in liver and skeletal muscle, catch up growth with age-matched CON, and significant metabolic differences in hunger and satiety hormones with a high-energy diet. Future studies are required to determine whether these alterations manifest as cardiometabolic disease as these animals age.

## Supplementary Material

1

## Figures and Tables

**Table 1.** Comparison between control (CON) and maternal undernutrition groups for morphometric measures at baseline and the end of the 7-week challenge

	Weight (Kg)			Height (cm)		BMI (Kg/cm2)	
Group	Newborn	0wk	7wk	0wk	7wk	0wk	7wk

CON	0.919	13.91	14.77	89.61	97.13	17.34	15.64

CON SEM	0.056	0.56	0.73	1.49	2.57	0.63	0.36

IUGR	0.727	11.41	12.65	88.67	93.75	14.37	14.20

IUGR SEM	0.043	1.00	1.20	2.69	2.31	0.68	0.66

p value 0wk IUGR vs CON	**0.022**	**0.024**	0.166	0.734	0.370	**0.001**	0.085

p value CON 7wk vs 0wk			**0.040**		**0.035**		0.072

p value IUGR 7wk vs 0wk			**0.015**		**0.011**		0.344

**Table 2.** Comparison between control (CON) and maternal undernutrition groups for body composition from DEXA at baseline and at the end of the 7-week challenge

	bone mass (gm)		fat mass (gm)		lean mass (Kg)		total mass (Kg)	
Group	0wk	7wk	0wk	7wk	0wk	7wk	0wk	7wk

CON	578.3	641.4	525.4	574.7	12.29	13.13	13.40	14.35

CON SEM	29.6	38.1	22.0	26.7	0.52	0.67	0.57	0.73

IUGR	468.5	550.2	406.7	479.8	9.57	11.22	10.44	12.25

IUGR SEM	56.7	69.5	40.5	52.6	0.94	1.20	1.04	1.32

p value 0wk IUGR vs CON	0.101	0.256	**0.021**	0.120	**0.023**	0.175	**0.025**	0.175

p value CON 7wk vs 0wk		**0.039**		**0.024**		**0.027**		**0.025**

p value IUGR 7wk vs 0wk		**0.012**		**0.017**		**0.016**		**0.0154**

**Table 3.** Comparison between control (CON) and maternal undernutrition groups for ALT, AST, glucose, and cortisol measures at baseline and at the end of the 7-week challenge (mg/dL)

Group	ALT (U/L)		AST (U/L)		Glucose (mg/dL)		Cortisol (ug/dL)	
	0wk	7wk	0wk	7wk	0wk	7wk	0wk	7wk

CON	21.43	11.43	26.14	24.43	61.57	61.00	39.94	38.31

CON SEM	4.02	2.64	1.92	4.36	1.99	1.91	2.32	4.10

IUGR	17.50	14.00	29.83	17.83	68.33	71.33	43.13	41.18

IUGR SEM	2.22	3.11	4.41	2.97	7.95	2.91	6.09	3.73

p value 0wk IUGR vs CON	0.433	0.539	0.435	0.253	0.394	**0.011**	0.613	0.620

p value CON 7wk vs 0wk		**0.004**		0.734		0.839		0.751

p value IUGR 7wk vs 0wk		0.328		**0.001**		0.791		0.795

**Table 4.** Comparison between control (CON) and maternal undernutrition groups for serum lipid measures at baseline and at the end of the 7-week challenge (mg/dL)

	HDL-C (mg/dl)		LDL-C (mg/dl)		Triglycerides (mg/dl)		TSC (mg/dl)	
Group	0wk	7wk	0wk	7wk	0wk	7wk	0wk	7wk

CON	66.21	55.21	32.29	30.29	35.86	46.29	99	85.64

CON SEM	2.55	3.37	3.31	2.78	2.82	5.19	4.12	5.16

IUGR	62.92	59.92	37.17	25.5	37.67	43.83	100.33	85.58

IUGR SEM	4.33	6.77	3.77	3.53	6.97	4.85	6.36	6.89

p value 0wk IUGR vs CON	0.534	0.534	0.350	0.303	0.804	0.739	0.860	0.995

p value CON 7wk vs 0wk		**0.029**		0.602		**0.039**		0.059

p value IUGR 7wk vs 0wk		0.643		**0.015**		0.394		**0.0054**

**Table 5.** Comparison between control (CON) and maternal undernutrition groups for serum insulin, leptin, and adiponectin at baseline and at the end of the 7-week challenge

Group	Adiponectin (ng/ml)		Adipsin (ng/ml)		Leptin (ng/ml)		Insulin (IU/mL)	
	0wk	7wk	0wk	7wk	0wk	7wk	0wk	7wk

CON	53,410.9	72,621.5	3658.2	5410.0	3.12	0.96	10.33	7.98

CON SEM	5977.1	18,754.5	249.7	401.0	0.35	0.35	2.69	2.09

IUGR	140429.0	85,664.6	4197.2	5860.2	5.36	2.51	31.68	14.71

IUGR SEM	36,809.1	15,178.8	324.3	632.9	0.50	0.44	17.20	3.06

p value 0wk IUGR vs CON	**0.042**	0.618	0.230	0.570	**0.005**	**0.023**	0.211	0.090

p value CON 7wk vs 0wk		0.280		**0.008**		**0.004**		0.522

p value IUGR 7wk vs 0wk		0.301		**0.030**		**0.020**		0.370

**Table 6.** Summary of comparison between control and maternal undernutrition groups for gene expression

	Adipose		Liver		Skeletal muscle	
	Chow	HFHS	Chow	HFHS	Chow	HFHS

Genes Up	1	13	194	6	49	48

Genes Down	0	20	53	8	35	93

Total Diff Exp	1	33	247	14	84	141

Genes Detected	19,419	19,419	19,419	19,419	19,419	19,419

Percent Up	100.0	39.4	78.5	42.9	58.3	34.0

Percent Down	0.0	60.6	21.5	57.1	41.7	66.0

Percent Diff	0.0	0.2	1.3	0.1	0.4	0.7

FDR corrected p-values, ≥1.2 fold change.

**Table 7.** Summary of comparison between control and maternal undernutrition groups for pathway enrichment

	Adipose		Liver		Skeletal muscle	
	Chow	HFHS	Chow	HFHS	Chow	HFHS

Pathways Up	0	1	31	19	3	2

Pathways Up EoP	–	0	5	0	1	0

Pathways Down	0	7	15	1	3	12

Pathways Down EoP	–	1	1	0	1	0

Total Diff Pathways	0	8	46	20	6	14

Percent Up	–	12.5%	67.4%	95.0%	50.0%	14.3%

Percent Down	–	87.5%	32.6%	5.0%	50.0%	85.7%

Pathway enrichment p-value < 0.01 for DEG p-value < 0.05 and contains FDR gene(s).

EoP - pathway met End of Pathway Criteria (see Methods).

**Table 8.** Summary of comparison between control and maternal undernutrition groups for regulatory networks

	Adipose		Liver		Skeletal muscle	
	Chow	HFHS	Chow	HFHS	Chow	HFHS

Networks Up	0	1	3	3	1	0

Percent Up-Regulated Genes	–	53	25	85	83	–

Networks Down	0	2	1	0	0	0

Percent Down-Regulated Genes	–	65	25	–	–	–

Total Diff Networks	–	3	4	3	1	0

Percent Networks Up	–	33.3%	75.0%	100.0%	100.0%	–

Percent Networks Down	–	66.7%	25.0%	0.0%	0.0%	–

Network p-value < 0.01 for direct connection genes < 0.05 and Network Regulator differentially expressed consistent with network activation/inhibition status.

**Table 9.** Comparison between control (CON) and maternal undernutrition groups for sugar drink consumption adjusted by body weight (L/Kg)

Group	1wk	2wk	3wk	4wk	5wk	6wk	7wk	7wk Avg
CON	0.053	0.054	0.047	0.035	0.045	0.035	0.009	0.040
CON SEM	0.015	0.007	0.011	0.008	0.011	0.012	0.006	0.005
IUGR	0.105	0.070	0.076	0.053	0.075	0.057	0.032	0.067
IUGR SEM	0.012	0.008	0.010	0.009	0.014	0.012	0.006	0.005
adj. p-value	**0.018**	0.155	0.062	0.411	0.199	0.300	0.108	**0.008**

**Table 10.** Summary of gene expression comparison between control (CON) and maternal undernutrition groups for HFCS vs chow diets

	Adipose		Liver		Skeletal muscle	
	CON	IUGR	CON	IUGR	CON	IUGR

Genes Up	8	1	1	295	195	7

Genes Down	0	0	8	614	137	27

Total Diff Exp	8	1	9	909	332	34

Genes Detected	19,419	19,419	19,419	19,419	19,419	19,419

Percent Up	100.0	100.0	11.1	32.5	58.7	20.6

Percent Down	0.0	0.0	88.9	67.5	41.3	79.4

Percent Diff	0.0	0.0	0.1	4.7	1.7	0.2

Includes FDR corrected p-values and ≥1.2 fold change.

**Table 11.** Summary of pathway enrichment comparison between control (CON) and maternal undernutrition groups for HFCS vs chow diets

	Adipose		Liver		Skeletal muscle	
	CON	IUGR	CON	IUGR	CON	IUGR

Pathways Up	0	0	1	57	14	3

Pathways Up EoP	–	–	0	0	1	1

Pathways Down	0	0	7	24	10	3

Pathways Down EoP	–	–	4	1	2	2

Total Diff Pathways	–	–	8	81	24	6

Percent Up	–	–	12.5%	70.4%	58.3%	50.0%

Percent Down	–	–	87.5%	29.6%	41.7%	50.0%

Pathway enrichment p-value < 0.01 for DEG p-value < 0.05 and contains FDR gene(s). EoP - pathway met End of Pathway Criteria (see Methods).

**Table 12.** Summary of regulatory network comparison between control (CON) and maternal undernutrition groups for HFCS vs chow diets

	Adipose		Liver		Skeletal muscle	
	CON	IUGR	CON	IUGR	CON	IUGR

Networks Up	1	1	0	0	2	0

Percent Up-Regulated Genes	61	50	–		60	–

Networks Down	0	1	7	4	2	1

Percent Down-Regulated Genes	–	47	82	55	46	98

Total Diff Networks	1	2	7	4	4	1

Percent Networks Up	100.0%	50.0%	0.0%	0.0%	50.0%	0.0%

Percent Networks Down	0.0%	50.0%	100.0%	100.0%	50.0%	100.0%

Network p-value < 0.01 for direct connection genes < 0.05 and Network Regulator differentially expressed consistent with network activation/inhibition status.

## Data Availability

The gene expression data of this study are openly available in repository Gene Expression Omnibus (GEO Series accession number GSE235713).
